# Determinants of stunting and severe stunting among Burundian children aged 6-23 months: evidence from a national cross-sectional household survey, 2014

**DOI:** 10.1186/s12887-017-0929-2

**Published:** 2017-07-25

**Authors:** Sandra Nkurunziza, Bruno Meessen, Jean-Pierre Van geertruyden, Catherine Korachais

**Affiliations:** 1 Global Health Institute, University of Antwerp, Gouverneur Kinsbergencentrum, Doornstraat 331–, -2610 Wilrijk, BE Belgium; 2 Health Community Department, University of Burundi, Boulevard du 28 Novembre, BP 1020 Bujumbura, Burundi; 3 Health Economics Unit, Department of Public Health, Institute of Tropical Medicine, Nationalestraat 155, 2000 Antwerp, Belgium

**Keywords:** Stunting, undernutrition, children, Burundi

## Abstract

**Background:**

Burundi is one of the poorest countries and is among the four countries with the highest prevalence of stunting (58%) among children aged less than 5 years. This situation undermines the economic growth of the country as undernutrition is strongly associated with less schooling and reduced economic productivity. Identifying the determinants of stunting and severe stunting may help policy-makers to direct the limited Burundian resources to the most vulnerable segments of the population, and thus make it more cost effective. This study aimed to identify predictors of stunting and severe stunting among children aged less than two years in Burundi.

**Methods:**

The sample is made up of 6199 children aged 6 to 23 months with complete anthropometric measurements from the baseline survey of an impact evaluation study of the Performance-Based financing (PBF) scheme applied to nutrition services in Burundi from 2015 to 2017. Binary and multivariable logistic regression analyses were used to examine stunting and severe stunting against a set of child, parental and household variables such as child’s age or breastfeeding pattern, mother’s age or knowledge of malnutrition, household size or socio-economic status.

**Results:**

The prevalence of stunting and severe stunting were 53% [95%CI: 51.8-54.3] and 20.9% [95%CI: 19.9-22.0] respectively. Compared to children from 6-11 months, children of 12-17 months and 18-23 months had a higher risk of stunting (AdjOR:2.1; 95% CI: 1.8-2.4 and 3.2; 95% CI: 2.8-3.7). Other predictors for stunting were small babies (AdjOR=1.5; 95% CI: 1.3-1.7 for medium-size babies at birth and AdjOR=2.9; 95% CI: 2.4-3.6 for small-size babies at birth) and male children (AdjOR=1.5, 95% CI: 1.4-1.8). In addition, having no education for mothers (AdjOR=1.6; 95% CI: 1.2-2.1), incorrect mothers’ child nutrition status assessment (AdjOR=3.3; 95% CI: 2.8-4), delivering at home (AdjOR=1.4; 95% CI: 1.2-1.6) were found to be predictors for stunting. More than to 2 under five children in the household (AdjOR=1.45; 95% CI: 1.1-1.9 for stunting and AdjOR= 1.5; 95% CI: 1.2-1.9 for severe stunting) and wealth were found to be predictors for both stunting and severe stunting. The factors associated with stunting were found to be applicable for severe stunting as well.

**Conclusion:**

Mother’s education level, mother’s knowledge about child nutrition status assessment and health facility delivery were predictors of child stunting. Our study confirms that stunting and severe stunting is in Burundi, as elsewhere, a multi-sectorial problem. Some determinants relate to the general development of Burundi: education of girls, poverty, and food security; will be addressed by a large array of actions. Some others relate to the health sector and its performance – we think in particular of the number of children under five in the household (birth spacing), the relationship with the health center and the knowledge of the mother on malnutrition. Our findings confirm that the Ministry of Health and its partners should strive for better performing and holistic nutrition services: they can contribute to better nutrition outcomes.

## Background

One of the sustainable development goals (SDGs) is to end all forms of malnutrition by 2030 [1]. There are two categories of malnutrition: on the one hand undernutrition which encompasses stunting, wasting and deficiencies of micronutrients (i.e. vitamins and minerals) and on the other hand overweight, obesity due to over-consumption of specific nutrients. Worldwide, in 2014, 23.8% of the children under-five years of age were stunted following the WHO definition, 7.5% were wasted but 6.1% had overweight or were obese [2, 3].

Undernutrition makes children more vulnerable to severe diseases. In 2015, undernutrition was considered to be an underlying contributing factor in about 45% of the 5.9 million children who died under the age of five. Actually, the number of global deaths and DALYs lost among children under-five years of age attributed to maternal and child undernutrition constitutes the largest percentage of all risks in this age group]. Moreover, child undernutrition is a strong predictor for less schooling and reduced economic productivity when adult [4, 5], which in turn are both risk factors for raising undernourished children, making it all a vicious circle. Thus, the fight against malnutrition is a long term investment for health but also for economic growth and social wellbeing for both present and future generations.

Developing countries host the bulk of the global stunting and child mortality rate. The situation is particularly critical in Sub-Sahara Africa where one third of the stunted under-five years of age children are retrieved and where stunted children are 14 times more likely to die before the age of five[6]. Actually, although the global trend in stunting has been decreasing from 39.6% in 1990 to 23.8% in 2014, the absolute number of stunted children in Africa has increased by 23% within the same period [3, 7]. This dramatic situation calls for actions; African leaders have to set up strategic plans to reduce both the epidemiologic and socioeconomic burden of malnutrition, and turn the vicious circle into a virtuous one.

**Fig. 1:**
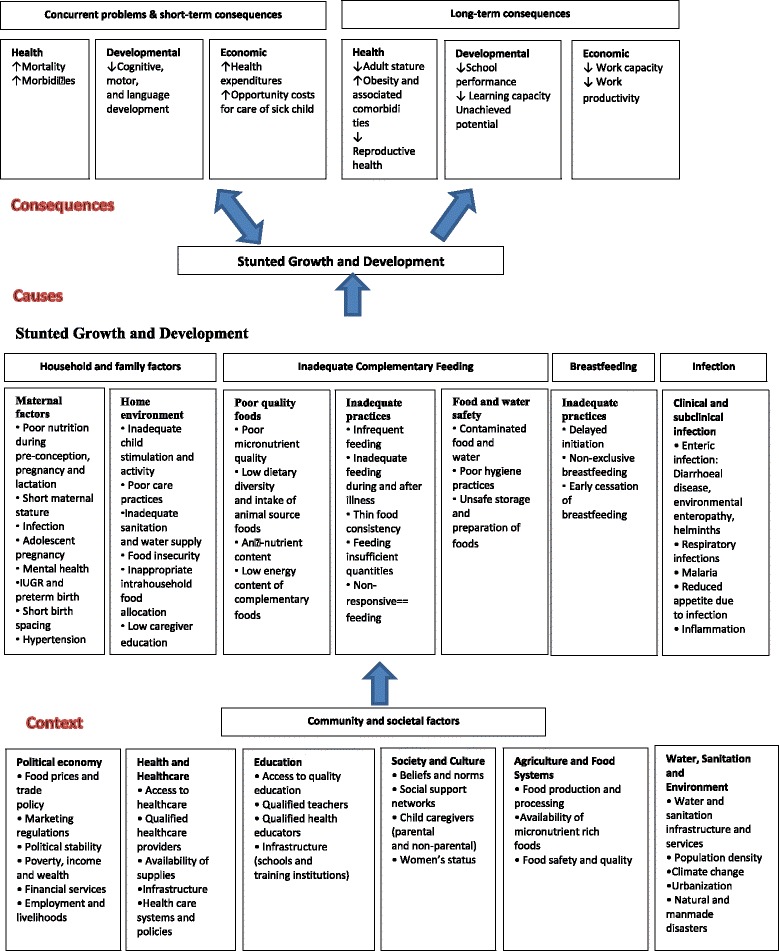
WHO conceptual framework on Childhood Stunting: Context, Causes, and Consequences

There is a large body of evidence on the factors of malnutrition in Low Income Countries (LICs) and sub-Saharan Africa. A multi-national cohort study revealed an association between poverty and stunting [8]. Suboptimal breastfeeding, and inappropriate complementary feeding practices, recurrent infections and micronutrient deficiencies are also important determinants of stunting [9, 10]. When poverty becomes an permanent condition, it leads to a cumulative inadequate food intake and poor health conditions from which arises stunting [11]: the increased frequency and severity of infections in poorly nourished children results in growth impairment[11]. More comprehensively, linear growth failure occurs within a complex interplay of other more distant community and societal factors, such as access to healthcare and education, political stability, urbanization, population density and social support networks: this has been described in the World Health Organization (WHO) Conceptual Framework on Childhood Stunting [12] (Figure 1).

This research zooms in on malnutrition in Burundi, one of the poorest countries in the world with an estimated per capita gross national income of $280 in 2013 [13]. Densely populated, it has a population of approximately 10.6 million inhabitants on a total area of 27,830 square kilometers and 90% of the population is living in rural areas from agriculture and 61.5% of the population in this area cannot meet their basic needs in terms of calorie intake [13]. Burundi has the highest prevalence of stunting (58%) worldwide, together with Timor Leste [14]. Burundian children aged less than five years suffer from an important mortality rate of 82‰ per year [15].

The available literature on the Burundian nutrition context consists mainly in reports from different partners in health looking at the trend of acute and chronic malnutrition in the most affected provinces of the country [16]. Beside those descriptive reports, there is an impact evaluation report of a nutrition program run in two provinces of eastern Burundi between 2010 and 2014. The two-year impact of the nutrition program consisting of three core components (distribution of food rations, participation in behavior change communication sessions delivered via care groups and attendance at preventive health services) had been positive on household access to food, child feeding practices and child morbidity. However, as the evaluation came too early in the study process, the impact on child undernutrition could not yet be evaluated [17–19]. A relevant report, in regards to our research, comes from UNICEF who used the 2010 Demographic and Health Survey data (DHS) to assess the predictors factors of child undernutrition in Burundi [20] and found that gender, age, mother’s age, wealth index, dependency ratio and region of residence were associated to stunting. Another study explored the impact of the civil war on child’s health status found, after controlling for province of residence, birth cohort, individual and household characteristics, and province-specific time trends, that children exposed to the war have on average 0.52 standard deviations lower height-for-age z-scores than non-exposed children [21].

We update and complete these findings to have a comprehensive knowledge about the determinants of stunting in the local Burundian context. This is vital to develop prevention strategies and strengthen nutrition intervention programs. We’ve included more independent variables such as mother’s knowledge, household’s food security, breastfeeding, birth weight proxy, place of delivery, arable land ownership. The findings should help policy-makers to direct the limited Burundian resources to the most vulnerable segments of the population, and thus make it more cost effective. It may also help in designing new intervention strategies aimed at reducing the number of malnourished children. Therefore, the aim of the study was to identify predictors of stunting and severe stunting among children aged less than two years in Burundi.

## Methods

### Study design and sample size

We used household baseline data from an impact evaluation study which aims to measure and understand the effects of the Performance-Based Financing (PBF) scheme applied to nutrition services in Burundi at facility level and community level. The protocol of this impact evaluation is described elsewhere [22]. Briefly, the study has a cluster-randomized controlled trial design, with health center as the primary unit of sampling and *sous-colline* (the smallest administrative entity with a variable number of villages) as the secondary unit sampling. The sample size was computed on the smallest difference in the main outcome that can be considered of public health significance which is equivalent to a reduction of ≅25% in acute malnutrition prevalence (2.5% points in absolute terms) in intervention centers as compared to control centers. Assuming that the intervention will decrease the prevalence of moderate acute malnutrition in children aged 6 to 23 months from 10% to 7.5% [23] while accepting a 2-sided α-error of 5% and a β-error of 20% indicated to survey at least 65 children aged 6-23 months in the catchment area of each health center. Among the 193 health centers providing nutritional services, 90 health centers were randomly selected (computer-based randomization) and randomized to either the intervention or control group. The number of children per health center was increased to 72 to allow for missing or incomplete data, amounting to a total of 6,480 children aged 6-23 months. The Nutrition PBF impact evaluation study is registered on ClinicalTrials.gov with the following identifier: NCT02721160 [22].

### Data Collection

Households were eligible for the survey when (i) they had at least one child aged 6-23 months and (ii) the eligible child was present together with their mother or primary caregiver and the household head. The first visited household was chosen as follows: from the center of the *sous-colline*, a pen was thrown in the air to indicate the direction to be taken by the surveyors; following this direction, the first household reached with an eligible child was the first to be surveyed (only if caregiver and head were present and gave their consent). The surveyors would then continue on the same direction to find the second household to be surveyed, and so on. In case of more than one eligible child in the household, one of them was randomly selected. Data collection tools consisted in three modules: a questionnaire administered to the household head, a questionnaire to the mother and one anthropometrics module. The household head questionnaire allowed to get information on general household characteristics such as household head education, gender and occupation, household size, distance to health center, as well as to assess the household socio-economic status and their food security status. The questionnaire administered to the mother collected information on her age, education, occupation and parity. It also allowed to get information on her feeding practices with the selected child and on her knowledge on nutrition; we also collected information on the health of the child (vaccination status, health problems in the last two weeks, visits to the health center). The module on anthropometrics collected the weight, height, mid-upper arm circumference and presence of edema of the child (as well as the mid-upper arm circumference (MUAC) of the mother).

In the field, surveyors worked in pairs with one supervisor per six pairs of surveyors. Each pair carried a SECA® 876 flat scale, a UNICEF measuring board and a SECA® 212 measuring tape. Surveyors were given comprehensive training in the taking of anthropometric measurements and a standardization exercise was carried out during the course of the training. The questionnaire was filled in on a smartphone, using the Open Data Kit Collect application[24], which allowed for: adding constraints into the data field, automatically skipping irrelevant questions/filtering to relevant questions, and obliging the surveyor to respond to every question before finalizing the questionnaire. Close supervision also allowed for a good quality control. Finally, lot quality assurance sampling (LQAS) was performed in order to ensure high quality anthropometrics measurements^1^.

### Data analysis

#### Stunting

We used the 2006 World Health Organization (WHO) Child Growth Standards. Height-for-age z-scores were used to assess the chronic nutritional status of children [25]. The height-for-age z-score expresses a child’s height in terms of the number of standard deviations (SDs) above or below the median height of healthy children in the same age group or in a reference group. Children with a measurement of <−2 SD from the median were considered as short for their age (stunted), while children with measurement of <−3 SD from the median group were considered to be severely stunted.

#### Explanatory variables

The explanatory variables were chosen on the basis of the WHO conceptual framework on childhood stunting [12] which is built on the UNICEF framework on causes of malnutrition (Figure 1). Both frameworks highlight the context, causes and consequences of stunting. However, the basic and underlying causes are more itemized in the WHO conceptual framework enabling a more context specific guidance in developing of nutrition-sensitive strategies.

We classified the factors into three levels: parental-, child-, household-level factors. Parental-level factors include maternal education, mother’s age, marital status as well as a variable assessing her knowledge of malnutrition. For the latter, we compared the mother’s satisfaction about the child’s nutrition status to the actual child’s nutrition status and categorized mothers with either a correct or an incorrect assessment of their child’s nutrition status.

Child-level factors were age, sex, place of delivery, child’s breastfeeding pattern, sickness episode within the two last weeks, feeding practices and a proxy of their birth weight. The age of children was estimated first by using the birth dates reported on their immunization card (94% of children) and only secondary by asking the mother.

In our survey sample, the birth weight was only present on the immunization card in 30% of the cases. It has been proven from 3 Demographic and Health Surveys (DHS) conducted in three low- and middle-income countries (LMICs) that the mother’s perception of size is a good proxy of birth weight [26] and in our study the 30% children of whom we knew the birth weight was also correlated (r=-0.44) with the mother’s perception. Therefore, we used the perceived size of the child at birth by the mother as a proxy of the child’s birth weight. Using the twenty-four hours recall on the child’s diet and based on the WHO guidelines on indicators assessing infant and young child feeding practices, we compute the minimum acceptable diet which encompasses the minimum dietary diversity and the minimum meal frequency [27].

Household level factors were household head education, food insecurity, socio-economic status, source of drinking water, time to the health center, household size and number of children aged less than 5 years in the household, arable land ownership. The assessment of household food insecurity was based on the 2007 Household Food Insecurity Access Scale (HFIAS) generic questions, created by the Food and Nutrition Technical Assistance (FANTA) project [28]. These have been validated in a number of different contexts and over different time-periods. The section includes nine occurrence questions, with an increasing level of severity of food insecurity (access) and nine questions concerning ‘frequency-of-occurrence’ to determine how often food insecurity occurred [28]. A household wealth index was calculated as a score of household assets such as ownership of means of transport, ownership of durable goods and household facilities. Weights for each variable were obtained thanks to a principal components analysis method [29]. This index was divided into five quintiles, and each household was assigned to one of these categories: poorest, poorer, middle, rich and richest.

### Statistical analyses

To determine the level of stunting and severe stunting in children aged 6-23 months, the dependent variable was expressed as a dichotomous, that is, “not stunted” (≥-2 SD) or “not severely stunted” (≥-3 SD) *versus* “stunted” (<-2 SD) or “severely stunted” (<-3 SD). Logistic regression analyses were performed using Stata® (version 12.1 College Station, Texas 77845 USA). Bivariate analysis was done for all explanatory variables to identify those associated with children stunting and severe stunting. Variables with p-value below 0.10 in the bivariate analysis were included in the multivariable analysis model. Adjustments for the cluster sampling design effects were incorporated using the “vce” command. A manual procedure of stepwise backward elimination process was then used to identify factors that were significantly associated with the study outcomes using 5 % significance level. The adjusted odds ratios (AdjOR) with 95% confidence Intervals (CIs) were calculated and those with *p*<0.05 were considered to be significant. Collinearity and interaction between independent variables were assessed.

## Results

### Characteristics of the sample

The respondent rate was 95.7% (n=6199). The prevalence of stunting and severe stunting were 53.0% (95% CI:51.8-54.3) and 21% (95% CI:19.9-22.0) respectively (Table 1). Male and female children were nearly equally represented as well as age categories. Among the children who experienced a sickness episode during the two last week 59.1% (95% CI:57.9-60.3), the majority had fever 54.6% (95% CI:53.0-56.7). 83.9% (95% CI:83.0-84.8) of the children were born at a health facility. Almost all children have been breastfed (99.9%; 95% CI:99.8-99.9) and 83.4% (95% CI:81.7-85.1) of the children aged between 18 and 23 months were still on breastfeeding at the moment of the survey. Only 24.8% (95% CI:23.9-26.0) of the children had the recommended diet according to their age with respect to frequency and diversity (Table 1).

**Table 1:** Characteristics of children aged 6–23 months: national cross-sectional survey, Burundi 2014

Child characteristics	Number	Stunted	Not stunted	Severely stunted	Not severely stunted	%[95% CI]
Nutrition status	6199					
Stunting		3291	2908	NA	NA	53.0% [51.8-54.3]
Severe stunting		NA	NA	1301	4898	20.9% [19.9-22.0]
Sex	6199					
Male		1747	1281	778	2250	48.8% [47.6-50.0]
Female		1544	1627	523	2648	51.1% [49.9-52.4]
Age (months)	6199					
6-11		843	1338	267	1914	35.1% [34.0-36.3]
12-17		1167	909	447	1609	33.4% [32.3-34.6]
18-23		1281	661	567	1375	31.3% [30.1-32.4]
Sickness episode within 2 weeks	6199					
No		1324	1207	485	2046	40.8% [39.6-42.0]
Yes	3668	1967	1701	816	2852	59.1% [57.9-60.3]
Diarrhea		727	556	315	968	34.9% [33.4-36.5]
Fever		1086	919	472	1533	54.6% [53.0-56.7]
Respiratory infection		617	600	264	953	33.1% [31.6-34.7]
Breastfeeding practices						
Has been breastfed	6197	3285	2906	1297	4895	99,9% [99.8-99.9]
Children weaned	6164	288	151	134	305	7.1% [6.4-7.7]
Exclusive 6 months breastfeeding	6074	2654	2308	1026	3936	81.6% [80.7-82.6]
Continuous to be breastfed						
6-11 months	2176	839	1331	264	1906	99.7% [99.5-99.9]
12-17 months	2067	1097	855	438	1514	94.44% [93.4-95.4]
18-23 months	1921	1046	557	453	1150	83.4% [81.7-85.1]
Minimum acceptable diet						
All	6144	834	701	316	1219	24.8% [23.9-26.0]
6-11months	2173	161	223	52	332	17.6% [16.0-19.2]
12-17 months	2060	310	258	120	448	27.5% [25.6-29.0]
18-23 months	1911	363	220	144	439	30.5% [28.4-32.5]
Place of delivery	6189					
Home		600	395	247	748	16.0% [15.1-16.9]
Health facility		2683	2511	1050	4144	83.9% [83.0-84.8]
Birth weight proxy (Mother’s perception on size of the baby at birth)	6174					
Large		579	767	191	1155	21.8% [20.7-22.8%]
Average		2157	1869	833	3193	65.2% [64.0-66.4]
Small		539	263	267	535	12.9% [12.1-13.8]

Half of the mothers were aged between 25 and 34 years. Around three quarters of them were without any education and two third had the impression that their babies were of average size at birth. The majority of the households visited were in couple (legally married or not) (91.9%; 95% CI:91.3-92.6).

At the moment of the interview, 58.5% (CI 95%:57.2-59.7) of the mothers perceived their children with a correct nutrition status (Table 2). Even though 90.3% (95% CI:89.6-91.1) of the households visited had arable land, 91.9% (95% CI:91.1-92.5) of them were experiencing food insecurity. The average household’s size was five persons (IQR=4-7). Half of the households (49.8%; 95% CI:48.5-51.0) lived at more than one hour walking to the health center (Table 3 ).

**Table 2:** Characteristics of the parents: national cross-sectional survey, Burundi 2014

Parental Characteristics	Number	Stunted children	Not stunted Children	Severely stunted children	Not severely stunted children	%[95 CI]
Mother’s education	6199					
No education		2449	2076	990	3535	73% [71.8-74.1]
Primary		765	684	283	1166	23.3% [22.3-24.4]
Secondary and above		77	148	28	197	3.6% [3.1-4.1]
Mother’s age	6081					
15-24 years		996	915	385	1526	31.4% [30.2-32.5]
25-34 years		1564	1427	620	2371	49.1% [47.9-50.4]
34-49 years		660	519	266	913	19.3% [18.3-20.3]
Mother’s child nutrition assessment *vs* current child’s nutrition status	6173					
Uncorrect		1799	764	593	1970	41.5% [4.29-42.7]
Correct		1478	2132	701	2909	58. 4% [57.2-59.7]
Marital status	6199					
In couple (legally married or not)		3012	2690	1165	4537	91.9% [91.3-92.6]
Live alone (div/sep/widow)		279	218	136	361	8.0%[7.3-8.6]

**Table 3:** Households’ characteristics: national cross-sectional survey, Burundi 2014

Household Characteristics	*n*	Stunted children	Not stunted children	Severely stunted children	Not severely stunted children	%[95 CI]
Household Size	6199					
<5		1752	1499	689	2562	52.4% [46.3-48.8]
≥5		1539	1409	612	2336	47.5% [46.3-53.6]
#Children Under 5	6199					
≤2		3103	2770	1127	4656	94.7% [94.1-95.3]
>2		188	138	84	242	5.2% [4.7-5.8]
Household head education	6015					
No education		2206	1843	916	3133	67.3% [66.1-68.5]
Primary		888	818	322	1384	28.3% [27.2-29.5]
Secondary		102	158	32	228	4.3% [3.8-4.8]
Arable land ownership	6195					
No		317	280	134	463	9.6% [8.9-10.3]
Yes		2972	2626	1166	4432	90.3% [89.6-91.1]
Source of drinking water	6199					
Not protected		1881	1618	746	2753	56.4% [55.2-57.6]
Protected		1410	1290	555	2145	43.5% [42.3-44.7]
Time to the Health centre	5996					
<30 min		654	651	244	1061	21.7% [20.7-22.8]
30-60 min		903	801	336	1368	28. 4% [27.2-29.5]
>60 min		1621	1366	681	2306	49. 8% [48.5-51.0]
Food security level	6189					
Food security		233	272	77	428	8.1% [7.4-8.8]
Low food insecurity		147	149	49	247	4.7% [4.2-5.3]
Moderate food insecurity		559	617	197	979	19.0% [18.0-19.9]
Severe food insecurity		2346	1866	974	3238	68.0% [66.8-69.2]

### Factors associated with stunting and severe stunting

#### Child level variables

Male was found to be associated with stunting (cOR=1.4; 95% CI: [1.3-1.5]; *p*<0.001) and severe stunting (cOR=1.7; 95% CI: [1.5-1.9]; *p*<0.001). The odds of being stunted for children aged 12 to 17 months and 18-23 months were respectively two times more (95% CI: [1.8-2.3] for stunted and 95% CI:[1.7-2.4] for severely stunted) and three times more (95% CI:2.7-3.4 for stunted and 95% CI:2.5-3.4 for severely stunted) than the odds of children aged 6-11 months (both *p*<0.001). Children aged 18-23 months for whom the minimum acceptable diet was correct in the 24 previous hours were less likely to be stunted (cOR=0.78; 95% CI: 0.64-0.96; *p*=0.02) and severely stunted (cOR=0.72; 95% CI: 0.58-0.91; *p*=0.005) than those from same age category with not appropriate complementary food. Children born at home were more likely to be stunted (cOR=1.4; 95% CI: 1.2-1.6; *p*<0.001) and severely stunted (cOR=1.3; 95% CI: 1.1-1.5; *p*=0.001) than those born at health facility.

#### Parental level variables

Children whose mothers had no education were more likely to be stunted (cOR=2.3; 95% CI: 1.7-3; *p*<0.001) and severely stunted (cOR= 2.0; 95% CI: 1.3-2.9; *p*<0.001) than those whose mothers reached secondary school and above. Children who were perceived by their mothers to be of medium or smaller size at birth were more likely to be stunted (cOR=1.5; 95% CI:1.3-1.7; *p*<0.001) (cOR=2.7; 95% CI:2.2-3.2; *p*<0.001) and severely stunted (cOR=1.5; 95% CI:1.3-1.8; *p*<0.001) (cOR=3.0; 95% CI:2.4-3.7; *p*<0.001) than those who were perceived to be larger. Children whose mother was not able to assess correctly the nutrition status were more likely to be stunted (cOR=3.4; 95% CI: 3.1-3.8; *p*<0.001) and severely stunted (cOR=1.2; 95% CI: 1.1-1.14; *p*<0.001) than those whose mother do know. Beside these common parental level factors associated with stunting and severe stunting in Burundian setting, the marital status of the mother (living in couple) was found to be associated with severe stunting (in couple: cOR:1.5; 95% CI: 1.2-1.8; *p*=0.001).

#### Household level variables

Children from a non-educated household head were more likely to be stunted (cOR=1.9; 95% CI: 1.4-2.4; *p*<0.001) and severely stunted (cOR=2.1; 95% CI: 1.4-3.0; *p*<0.001) than children from household head with secondary school and above. Children who were living at more than one walking hour from the health center had 1.2 (95% CI: 1.1-1.1.3; *p*: 0.01) times more odds to be stunted and 1.2 (95% CI: 1.1-1.5; *p*: 0.003) times more odds to be severely stunted than those living at less than 30 min walking. Children from household experiencing severe food insecurity had 1.4 (95% CI: 1.2-1.7; *p*<0.001) more odds of stunting and 1.6 (95% CI: 1.3-2.1; *p*<0.001) more odds of severe stunting than those living in food secured household. Children from poor households were more likely to be stunted compared to all other wealthier categories. Beside these common household level factors associated with stunting and severe stunting, the number of children under five of years in the household was found to be associated with severe stunting (Table 4).

**Table 4:** Factors associated with stunting and severe stunting in Burundian children aged 6-23 months, 2014

	Stunted				Severely stunted			
Child characteristics	Crude OR	*p*-value	Adjusted OR	*p*-value	Crude OR	*p*-value	Adjusted OR	*p*-value
Sex								
Female	1.0		1.0		1.0		1.0	
Male	1.4 [1.3-1.5]	<0.001	1.5 [1.4-1.8]	<0.001	1.7 [1.5-1.9]	<0.001	1.9 [1.6-2.2]	<0.001
Age (months)								
6-11	1.0		1.0		1.0		1.0	
12-17	2.0 [1.8-2.3]	<0.001	2.1 [1.8-2.4]	<0.001	2.0 [1.7-2.4]	<0.001	2.2 [1.9-2.6]	<0.001
18-23	3.0 [2.7-3.4]	<0.001	3.2 [2.8-3.7]	<0.001	2.9 [2.5-3.4]	<0.001	3.0 [2.7-3.9]	<0.001
Sickness episode within 2 weeks								
No	1.0				1.0			
Yes	1.0 [0.9-1.1]	0.31			1.2 [1.1-1.4]	0.003		
Place of delivery								
Health facility	1.0		1.0		1.0			
Home	1.4 [1.2-1.6]	<0.001	1.4 [1.2-1.6]	<0.001	1.3 [1.1-1.5]	0.001	1.2 [1.1-1.5]	0.03
Exclusive 6 months breastfeeding								
No	1.0				1.0			
Yes	1.1 [0.9-1.2]	0.20			0.90 [0.77-1.06]	0.20		
Continuous to be breastfed								
6-11 months								
No	1.0				1.0			
Yes	1.2 [0.2-6.9]	0.27			0.69 [0.08-5.95]	0.74		
12-17 months								
No	1.0				1.0			
Yes	1.0[0.7-1.4]	0.11			0.90 [0.57-1.39]	0.63		
18-23 months								
No	1.0				1.0			
Yes	0.81 [0.62-1.05]	0.12			0.80 [0.62-1.03]	0.09		
Minimum acceptable diet								
All								
No	1.0				1.0			
Yes	1.1 [0.9-1.2]	0.25			0.97 [0.84-1.12]	0.68		
6-11 months								
No	1.0				1.0			
Yes	1.1 [0.9-1.4]	0.15			1.1 [0.8-1.6]	0.38		
12-17 months								
No	1.0				1.0			
Yes	0.91 [0.75-1.11]	0.37			0.89 [0.70-1.12]	0.33		
18-23 months								
No	1.0				1.0			
Yes	0.78 [0.64-0.96]	0.02			0.72 [0.58-0.91]	0.005		
Birth weight proxy (mother’s perception of the baby size at birth)								
Large	1.0				1.0		1.0	
Medium	1.5 [1.3-1.7]	<0.001	1.5 [1.3-1.7]	<0.001	1.5 [1.3-1.8]	<0.001	1.6 [1.3-1.9]	<0.001
Small	2.7 [2.2- 3.2]	<0.001	2.9 [2.4-3.6]	<0.001	3.0 [2.4-3.7]	<0.001	3.3 [2.6-4.1]	<0.001
Parental characteristics								
Maternal education								
Secondary and above	1.0		1.0		1.0			
Primary	2.1 [1.6-2.9]	<0.001	1.6 [1.2-2.1]	0.002	1.7 [1.1-2.6]	0.01		
No education	2.3 [1.7-3]	<0.001	1.6[1.2-2.1]	0.001	2.0 [1.3-2.9]	<0.001		
Mother’s age								
15-24 years	1.0				1.0			
25-34 years	1.0 [0.9-1.1]	0.91			1.0 [0.9-1.2]	0.62		
34-49 years	1.1 [1.0-1.3]	0.03			1.1 [0.9-1.3]	0.11		
Mother’s nutrition assessment *vs* current child’s nutrition status								
Correct	1.0				1.0			
Uncorrect	3.4 [3.1-3.8]	<0.001	3.3 [2.8-4]	<0.001	1.2 [1.1-1.14]	<0.001		
Marital status								
Live in couple ( married or not) Live alone (div/sep/widow)	1.0				1.0			
Live alone (div/sep/widow)	1.1 [0.95-1.4]	0.10			1.5 [1.2-1.8]	0.001		
Household characteristics								
Household head education								
Secondary and above	1.0				1.0			
Primary	1.7 [1.3-2.2]	<0.001			1.7 [1.1-2.4]	0.01		
No education	1.9 [1.4-2.4]	<0.001			2.1 [1.4-3.0]	<0.001		
Household Size								
<5	1.0				1.0			
>=5	1.0 [0.9-1.1]	0.1			1.0 [0.9-1.1]	0.6		
#Children Under 5								
1 or 2	1.0		1.0		1.0		1.0	
>2	1.2 [0.97-1.5]	0.08	1.45 [1.1-1.9]	0.003	1.3 [1.1-1.17]	0.03	1.5 [1.2-1.9]	0.001
Time to the Health centre								
<30 min	1.0				1.0			
30-60 min	1.1 [0.9-1.3]	0.1			1.0 [0.8- 1.2]	0.4		
>60 min	1.2 [1.1- 1.3]	0.01			1.2 [1.1- 1.5]	0.003		
Arable land ownership								
Yes	1.0				1.0			
No	1.0 [0.84-1.2]	0.99			1.1 [0.90-1.3]	0.36		
Source of drinking water								
Protected	1.0				1.0			
unprotected	1.1 [0.96-1.2]	0.23			1.0 [0.93-1.2]	0.46		
Food security level								
Food secure	1.0				1.0			
Low food insecure	1.1 [0.8-1.5]	0.34			1.1 [0.7-1.6]	0.62		
Moderate food insecure	1.0 [0.8-1.3]	0.60			1.1 [0.8-1.4]	0.44		
Severe food insecure	1.4 [1.2- 1.7]	<0.001			1.6 [1.3- 2.1]	<0.001		
SE status								
Richest	1.0		1.0		1.0		1.0	
Richer	1.4 [1.2-1.7]	<0.001	1.2 [1.1-1.5]	0.01	1.4 [1.1-1.7]	0.003	1.3 [1.1-1.7]	0.03
Middle	1.7 [1.5-2.0]	<0.001	1.5 [1.21.7]	0.001	1.8[1.4-2.2]	<0.001	1.6 [1.3-2.1]	<0.001
Poor	2 [1.6-2.3]	<0.001	1.7 [1.4-2.1]	<0.001	2 [1.6-2.5]	<0.001	1.9 [1.5-2.4]	<0.001
Poorest	2.1[1.8-2.4]	<0.001	2 [1.6-2.3]	<0.001	2.4 [1.9-2.9]	<0.001	2.4 [1.9-2.9]	<0.001

### Predictors for stunting

Male children were more likely to be stunted than girls (AdjOR=1.5; 95% CI: 1.4-1.8; *p*<0.001) (Table 4). Increasing age was associated with stunting (AdjOR=2.1; 95% CI: 1.8-2.4; *p*<0.001 for children aged 12-17 months and AdjOR=3.2; 95% CI: 2.8-3.7; *p*<0.001 for children aged 18-23 months). Children who were perceived by their mothers to be of medium or smaller size at birth were more likely to be stunted than those who were perceived to be larger (AdjOR=1.5; 95% CI: 1.3-1.7; *p*<0.001) (AdjOR=2.9; 95% CI: 2.4-3.6; *p*<0.001). Children who were delivered at home were more likely to be stunted (AdjOR=1.4; 95% CI: 1.2-1.6; *p*<0.001) and severely stunted (AdjOR=1.2; 95% CI: 1.1-1.5; *p*=0.03).

Children whose mothers had no schooling were more likely to be stunted compared with children whose mothers attained secondary school or above (AdjOR=1.6; 95% CI: 1.2-2.1; *p*=0.001). Children whose mother uncorrectly assess the nutrition status were more likely to be stunted than those whose mother do (AdjOR=3.3; 95% CI: 2.8-4; *p*<0.001). Children who were delivered at home were more likely to be stunted (AdjOR=1.4; 95% CI: 1.2-1.6; *p*<0.001). Being in a household with more than two under five years children was associated with more risk of being stunted than being in a household with one or two under five years children (AdjOR=1.4; 95% CI: 1.1-1.9; *p*=0.003). Children from poorest households were more likely to be stunted compared to all other categories (AdjOR=2; 95% CI: 1.6-2.3; *p*<0.001) (Table 4).

### Predictors for severe stunting

Age was significantly associated with severe stunting (AdjOR=2.2; 95% CI: 1.9-2.6; *p*<0.001 for children aged 12-17 months and AdjOR=3.0; 95% CI: 2.7-3.9; *p*<0.001 for children aged 18-23 months compared to children aged 6 to 11 months) and male children were more likely to be severely stunted than females (AdjOR=1.9; 95% CI: 1.6-2.2; *p*<0.001) (Table 4). Children who were perceived by their mothers to be of medium or smaller size at birth were more likely to be severely stunted than those who were perceived to be larger (AdjOR=1.6; 95% CI: 1.3-1.9; *p*<0.001) (AdjOR=3.3; 95% CI: 2.6-4.1; *p*<0.001). Living in a household with more than two under five years children was associated with more odds of being severely stunted than living in a household with one or two under five years children (AdjOR =1.5; 95% CI: 1.2-1.9; *p*=0.001). Children from poorest households were more likely to be severely stunted compared to all other categories (AdjOR=2.4; 95% CI: 1.9-2.9; *p*<0.001) (Table 4).

Inappropriate complementary feeding practices was correlated with household socio-economic status (*r*=0.1) and household food security level (*r*=-0.2). The latter two were also found to be correlate (*r*=-0.3).

Collinearity was assessed and found for sanitation and source of drinking water. There was no interaction between independent variables.

## Discussion

The prevalence of stunting and severe stunting among children aged 6 to 23 months was 53.0% and 21% respectively. These figures are similar to those from the last DHS conducted in 2010 (58.0%) [23]. This prevalence is high compared to the estimated prevalence of stunted pre-school children for the UN regions and sub-regions in 2015 [7]. Beside the heavy burden in terms of lost DALYS, international studies have shown that undernutrition is strongly associated with less schooling on the medium term [4, 5], and reduced economic productivity on the long term [30], something has to be done in order to stop the vicious circle.

Our study showed that the increased age of the child was associated with stunting and severe stunting. Similar findings were reported in other studies conducted in different LMICs [31, 32]. This could be explained by the inappropriate complementary food that children receive, due to the high prevalence of household’s severe food insecurity (68%). As children are growing up, they need adequate complementary food, in quantity and in quality, as a complement to the breast milk [10, 33]: our study found that only 30% of the children aged between 18-23 months received appropriate complementary food in regards to both frequency and diversity. In the bivariate analyses, stunting and severe stunting were associated with inappropriate complementary feeding practices but this turned out to be non-significant in the multivariate analysis.

Gender was another predictor of stunting and severe stunting in children aged 6-23 months as boys had higher odds of becoming stunted or severely stunted compared to girls, supporting previous findings in the region [31, 34]. A meta-analysis of 16 demographic and health surveys in Sub-Saharan Africa revealed the same results with an explanation oriented towards a historical pattern of preferential treatment of females due to high value placed on women’s agricultural labor [35]. However, such hypothesis cannot be ascertained in our study as there is a gender balance among children who had appropriate complementary food in the previous 24 hours before the survey.

Children whose mothers perceived them to be small or medium size at birth-a proxy of birth weight- were found to be at higher odds of being stunted and severely stunted compared to those perceived to be larger. Other studies conducted in Tanzania in 2015 and in Nepal in 2014 had the same findings [31, 36]. More recently, a cohort study conducted in Benin confirms that low birth weight was associated with growth impairment [37].

Our study also reveals that mothers assessing correctly the child nutrition status were less likely to have stunted children than those who did not assess this correctly. This could let assume that mothers who reserved time to learn how to evaluate child nutrition status are the ones who invest in the latter.

Children whose mothers reached secondary school and above were less likely to be stunted than those whose mothers had no schooling which also have been shown in previous studies elsewhere [33]. These two findings demonstrate the importance of education of girls as one strategy to overcome the burden of stunting and to promote good feeding practices for young children. In the present study, children who were delivered at health facility were less likely to be stunted compared to those delivered at home. This matches with findings from the Tanzanian (2015) and Kenyan (2012) studies [31, 34]. This finding hypotheses that mothers who delivered at health facility often use health services, and are thus more informed about good child health care practices.

Children belonging to households with more than two under five children were more likely to be stunted or severely stunted than the others. These results are similar to those found in Somalia [38]. Indeed, more under five children in the family may lead to a higher risk of having insufficient complementary food in a context of severe food insecurity. The children from the poorest socio-economic class were more likely to be stunted and severely stunted than all other categories. This finding is also supported by different studies conducted in LMIC [32, 39].

The factors that our study revealed to be predictors for stunting and severe stunting can be classified according the WHO conceptual framework on childhood stunting 12] into household and family factors (maternal factors and home environment) such as: maternal education, mother’s nutrition status assessment, number of under five years children in the household, size at birth. The others are found to be predictors that are reflected in the context in which the child lives: socio-economic status, place of delivery. As mentioned above, these findings are supported by other studies done elsewhere.

### Strengths and weaknesses of the study

The big size of our sample makes our findings precise and reliable for the whole population of the rural parts of Burundi. Though we cannot extrapolate the findings to other countries, they however suggest some trends in similar settings. However since the study has been conducted in rural areas, our findings are not applicable as such for urban settings.

We considered many more variables than previous studies, such as mother’s knowledge on child‘s nutrition status assessment, household’s food security, birth weight proxy, place of delivery, arable land ownership.

A weakness from the surveys was that any reading note wasn’t submitted to the interviewee to make sure of his ability to read and write: this didn’t allow us to consider the literacy variable for the analysis. Another weakness is that we didn’t push further to investigate community and societal factors specifically political economy factors and health factors of the WHO conceptual framework on childhood stunting.

### Implications for future research

The study suggests possible new paths for action which could trigger different action research projects, through nutrition community interventions, sensitization for family planning, young girls education, etc. As for the impact of performance-based financing as a mean to reduce malnutrition, through e.g. support nutrition community interventions, we will wait for the follow-up household survey (due early 2017) to perform the impact analyses. Possible other paths for action to study would be better targeting (e.g. through a demand-side financing scheme focused on poorest households), and nutrition community interventions (e.g. sensitization).

## Conclusion

We observed that child’s age, boys, a small birth weight (as perceived by the mother), more than two children under-five years of age in the household and household’s poor socioeconomic status were factors associated with stunting and severe stunting. Modeling selected that, mother’s education level, mother’s knowledge about child nutrition status assessment and health facility delivery were predictors of child stunting.

Our study confirms that stunting and severe stunting is in Burundi, as elsewhere, a multi-sectoral problem. Some determinants relate to the general development of Burundi: education of girls, poverty, and food security; will be addressed by a large array of actions. Some others relate to the health sector and its performance – we think in particular of the number of children under five in the household (birth spacing), the relationship with the health center and the knowledge of the mother on malnutrition. Our findings confirm that the Ministry of Health and its partners should strive for better performing and holistic nutrition services: they can contribute to better nutrition outcomes.

## Abbreviations

AdjOR: Adjusted Odds Ratio
cOR: Crude Odds Ratio
DHS: Demographic and Health Survey
FANTA: Food and Nutrition Technical Assistance
GDP: Gross Domestic Product
HFIAS: Household Food Insecurity Access Scale
IRB: Institutional Review Board
LMIC: Low and Middle Income Country
NCT: National Clinical Trial
PBF: Performance Based-Financing
SD: Standard Deviation
SDG: Sustainable Development Goals
UNICEF: United Nations of International Children's Emergency Fund
WHO: World Health Organization.

## Footnotes

1 Twice a week, external supervisors followed randomly chosen surveyors and measured again the weight, height, MUAC and oedema presence among the surveyed children; their measurements were confronted to the ones performed by the surveyors and errors were discussed with them. Observed discrepancies were null on average and big discrepancies (more than 200g for weight and 5mm for height and MUAC) were rare which tend to confirm that anthropometrics were of good quality.
